# Facile Synthesis of a Series of Non-Symmetric Thioethers Including a Benzothiazole Moiety and Their Use as Efficient In Vitro anti-*Trypanosoma cruzi* Agents

**DOI:** 10.3390/molecules24173077

**Published:** 2019-08-24

**Authors:** Alcives Avila-Sorrosa, Jazz D. Tapia-Alvarado, Benjamín Nogueda-Torres, Karla Fabiola Chacón-Vargas, Francisco Díaz-Cedillo, María Elena Vargas-Díaz, David Morales-Morales

**Affiliations:** 1 Instituto Politécnico Nacional, Escuela Nacional de Ciencias Biológicas, Departamento de Química Orgánica, Carpio y Plan de Ayala S/N, Colonia Santo Tomás, 11340 Ciudad de México, México; 2 Instituto Politécnico Nacional, Escuela Nacional de Ciencias Biológicas, Departamento de Parasitología, Carpio y Plan de Ayala S/N, Colonia Santo Tomás, 11340 Ciudad de México, México; 3 Instituto de Química, Universidad Nacional Autónoma de México, Circuito Exterior s/n, Ciudad Universitaria, C.P. 04510, Ciudad de México, México

**Keywords:** *Trypanosoma cruzi*, Chagas disease, benzothiazole derivatives, non-symmetric thioethers, neglected diseases

## Abstract

A series of 2-benzylsulfanyl benzothiazole (BTA) derivatives were synthesized and fully characterized and in vitro tested against two strains of *T. cruzi* (NINOA and INC-5), exhibiting good activities at low concentrations.

## 1. Introduction

The benzothiazole (BTA) is a heterocyclic compound of great interest in different areas of chemistry, materials, and biological sciences. The BTA core is rarely found in nature (e.g., luciferin, marine natural compounds, etc.); thus, most of the known derivatives have been prepared by chemical synthesis. Nevertheless, compounds including these structures have found applications in the synthesis of polymers, dyes, chemical sensors, herbicides, etc. In addition, they represent a wide variety of important biologically active xenobiotics, including antidiabetic [1], anticonvulsant agent [2], anticancer [3], anti-inflammatory [4], and antiparasitic [5]. Thus, this privileged pharmacological unit is one of the "master keys" for the design and synthesis of bioactive compounds with applications in medicinal chemistry and pharmaceuticals.

Several reports have shown that functionalization of the BTA system on position 2 is key for their enhanced biological activity mainly as antimicrobial agents. Thus, there is currently a growing interest in obtaining 2-substituted BTAs to direct them as significant active antiparasitic agents [6,7,8,9,10].

In this line, American trypanosomiasis or Chagas disease is caused by the hemoflagellate protozoan *Trypanosoma cruzi* (*T. cruzi*) that is transmitted by vectors, mainly triatomine insects [11]. This parasitic infection is considered neglected and affects mainly countries of tropical zones of Americas, where it is estimated that between 6 to 7 million people are infected with *T. cruzi* [12], and approximately 100 million people are at risk of infection with this protozoan [13]. Due to the increased number of cases of infections registered in recent decades in non-endemic areas, such as the United States, Canada, and European countries [14], Chagas disease is a serious emerging public health problem worldwide.

Clinically, Chagas disease occurs in acute and chronic phases. The first can be asymptomatic or manifest with fever or edema, or both, on the face and extremities. This phase is characterized by the presence of many parasites in the trypomastigote stadium circulating through the bloodstream, while in the chronic phase, there is a lower blood parasitaemia, but manifestations of the disease are most serious, leading to heart problems, megaviscera, neurological changes, and sudden death [15].

**Figure 1:**
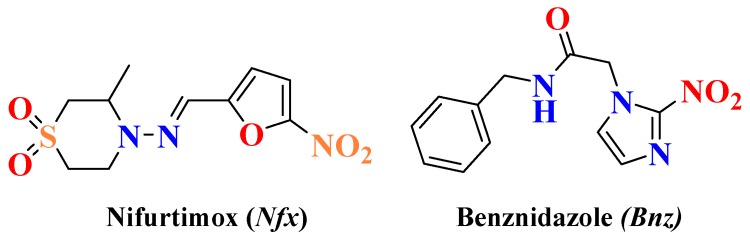
Main antichagasic chemotherapeutic agents.

Regarding treatment, there are only two nitrated chemotherapeutic agents: nifurtimox (Nfx) ((*R,S*)-3-methyl-*N*-[(1*E*)-(5-nitro-2-furyl)methylene]thiomorpholin-4-amine-1,1-dioxide) and benznidazole (Bnz) (*N*-benzyl-2-(2-nitro-1*H*-imidazol-1-yl)acetamide) (Figure 1), which are usually more effective in the acute stage. However, therapy with both drugs shows serious side effects, as well as the development of resistance to parasites [16].

Thus, based on the above, we would like to report the facile synthesis of a series of non-symmetric thioethers, including a BTA moiety, and their use as efficient in vitro anti-*Trypanosoma cruzi* agents.

## 2. Results and Discussion

### 2.1. Synthesis

**Scheme 1:**
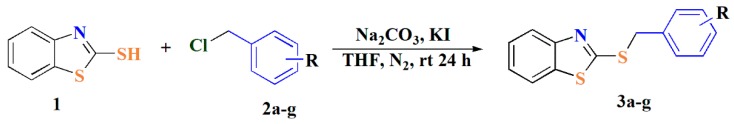
Synthesis of 2-benzylsulfanyl-BTA derivatives.

The structures of the series of BTA derivatives obtained are shown in Table 1. Their synthesis was achieved in a facile, single step procedure from the stoichiometric reaction of 2-mercaptoBTA **1** with different benzyl chlorides (**2a**–**g**), according to Scheme 1. Affording derivatives **3a**–**g** (yields ranging from 75 to 87%) as microcrystalline solids (derivatives **5** and **6** were obtained as liquids) with melting points lower than 100 °C (Table 1).

Analysis of compounds 2-(benzylthio)benzo[d]thiazole (**3a**) [17], 2-(4-chlorobenzylthio)benzo[d]thiazole (**3b**) [18], 2-(4-methoxybenzylthio)benzo[d]thiazole (**3c**) [19], 2-(4-fluorobenzylthio)benzo[d]thiazole (**3d**) [17], and 2-(3-fluorobenzylthio)benzo[d]thiazole (**3e**) [20] are coherent with those reported in the literature.

#### Spectroscopic Characterization

The series of compounds were characterized by spectroscopic methods (IR, NMR), mass spectrometry (EM-ESI), and elemental analysis. In general, the IR vibration analysis shows the main diagnostic signals for the different BTA derivatives. Hence, C-H stretching bands are observed in the range of ν 3080 to 2840 cm^−1^ due to the aromatic and aliphatic hydrocarbon units. Between ν 1610 and 1360 cm^−1^, a series of sharp and intense bands due to the vibrations of the BTA moiety are observed. In the case of the fluorinated compounds, a very intense signal around ν 990 cm^−1^ due to the C-F stretch is observed. Mass spectrometry analysis by electrospray ionization showed, in all cases, a single peak in the corresponding spectra due to the molecular ion plus one mass unit (M^+^ + 1).

Further, analysis by ^1^H, ^13^C{^1^H}, and ^19^F{^1^H} NMR afforded spectra exhibiting a number of signals on the proper chemical shifts coherent with the proposed structures of the series of BTAs. Thus, ^1^H NMR spectra showed signals in the range of 7.84–7.30 ppm due to the protons of the BTA moiety (4H). In addition, a diagnostic signal due to the benzylic protons (2H) was found at about 5 ppm. Moreover, the spectra of the BTA derivatives having a *para*-substituted benzene ring, exhibited a typical set of signals corresponding to an AA’BB’ system (4H) in the aromatic zone between 7.9 and 6.7 ppm. Besides, the ^13^C{^1^H} NMR spectra exhibited the seven expected signals for the BTA fragment in the range of 135–110 ppm. The signal due to the benzylic carbon was observed between 24–36 ppm. In the case of the fluorine-bonded carbons, these exhibited coupling constants of *J* = 244 Hz, this value being characteristic for this type of couplings. Finally, the ^19^F{^1^H} NMR spectra obtained were consistent with the different fluorine substituted derivatives **3d**–**g**.

### 2.2. Biological Evaluation

As mentioned above, the acute phase of American trypanosomiasis is characterized by a high parasitaemia and bloodstream presence trypomastigotes, these parasitic forms are highly infectious for myocardial cells and enterocytes among others, where they can remain for years as intracellular amastigotes resulting in the chronic phase of the disease [21]. Thus, given the fact that parasites on the bloodstream trypomastigotes stadium are the infective form, in mammals it becomes important the performing of trypanocidal evaluation of new compounds considering at least two or more strains endemic in the region.

In this line, the trypanocidal activity of the series of 2-benzylsulfanyl-BTA derivatives was evaluated in vitro against bloodstream trypomastigotes of two strains of *T. cruzi* endemic in Mexico (Table 2). Hence, different concentrations were evaluated to determine the LC_50_ of each compound. Additionally, the cytotoxic effect and the selectivity index (SI) were determined. Table 2 shows the results obtained for each of the compounds, as well as the reference drugs employed.

From Table 2, it can be observed that compounds **3a**, **3f,** and **3g** exhibited trypanocidal activity in bloodstream trypomastigotes of *T. cruzi* NINOA, with lower values of LC_50_ than Bnz (LC_50_ = 173.46 μM), and having compound **3f** as the best of this group (LC_50_ = 109.76 μM) exhibiting similar activity to that of Nfx (LC_50_ = 96.96 μM). However, when the evaluation was performed on trypomastigotes of *T. cruzi* INC-5, compound **3g** (LC_50_ = 185.35 μM) exhibited the best trypanocidal effect when compared with Bnz (LC_50_ = 216.57 μM). In addition, compounds **3b** (LC_50_ = 262.62 μM), **3c** (LC_50_ = 275.67 μM) and **3f** (LC_50_ = 259.81 μM) exhibited similar activities, however, still better that those observed with both reference drugs, being only closer to that produced by Bnz. Finally, compound **3b** only showed activity against *T. cruzi* INC-5, while compounds **3d** and **3e** showed null activity in neither of both strains. None of the synthesized compounds exceeded the activity of Nfx in both strains.

Interestingly, when the cytotoxic effect in macrophages was studied, six of the seven BTA derivatives evaluated exhibited a reduced cytotoxic effect on mammalian cells, compared with both reference drugs. Hence, compounds **3a**, **3b**, **3c**, **3d,** and **3e** were over two times less toxic than Nfx and about three-fold less toxic than Bnz. Compounds **3d** and **3e** were not considered in this analysis because they did not exhibit trypanocidal activity. Moreover, although compound **3g** resulted to be the most toxic compound of the series, it is still slightly less toxic than Bnz.

As a consequence of the combination of trypanocidal effect and low toxicity observed with compounds **3a**, **3b**, **3c,** and **3f,** their SI were better. Thus, all active compounds exhibited better SI values than Bnz in both strains. In the case of Nfx, its SI value was surpassed by the values exhibited by compounds **3a** and **3f** on *T. cruzi* NINOA and by compounds **3a** and **3c** on *T. cruzi* INC-5. However, although compound **3b** produced the best SI values for *T. cruzi* INC-5, the most interesting compounds of the series are **3a** and **3c**, since they were the only species showing trypanocidal activity in both strains with minimum cytotoxic effect. Thus, these compounds are the best candidates for further studies since they have similar or better selectivity than that of the reference drugs.

The bloodstream trypomastigotes of *T. cruzi* NINOA were more sensitive to both series of BTA and reference drugs, requiring lower concentrations to reach the 50% lysis of the parasites in comparison with those of the *T. cruzi* INC-5 string. This is probably due to the different origin of both strains because the *T. cruzi* NINOA strain was isolated form a patient with acute Chagas, while the *T. cruzi* INC-5 strain was obtained from a chronic Chagas patient. Besides, it is well known that among the *T. cruzi* strains, there is a wide genetic diversity, and thus their biological behavior may vary [22].

Interestingly, structure-activity relationship analysis (SAR) showed that the amount of fluorine in the BTA molecules plays an important role on their trypanocidal activity. Thus, compounds **3d** and **3e** did not show lytic activity on either of the strains, having only one fluorine on their structures, and their trypanocidal activity is low in comparison with that observed, in both strains, for compounds **3f** and **3g**, which have two or more fluorines on their structures. In addition, compound **3b** has a single chlorine proved to be active with the *T. cruzi* INC-5 strain, however, not with *T. cruzi* NINOA strain.

On the other hand, compounds **3a** and **3c**, which do not have halogens on their structures, only exhibited moderated trypanocidal activity. While derivatives **3a**, **3b**, and **3c** were the less cytotoxic (Table 2). Noteworthy is the fact that cytotoxicity increases for those BTA’s with fluorines on their structures, with a clear trend of increase in their cytotoxicity as the number of fluorines on their structures increases, i.e., **3g** > **3f** > **3e** > **3d**.

Improving selectivity in drug design is a must. Thus, from the series of BTA’s compounds attained, it was important to determine whether their biological activity was a consequence of being selective or due to their general toxicity [23]. Thus, results from the experiments showed that in the case of compounds **3f** and **3g** their trypanocidal effect is due to toxicity and not to selectivity. Meanwhile, those compounds without halogens on their structures, i.e., **3a** and **3c**, exhibited the best SI indexes (Table 2) for both strains.

In summary, we synthesized, in a facile manner, a series 2-substituted-BTA’s, that exhibited good trypanocidal activity at low concentrations, thus being promising candidates for further, more detailed studies in both in vitro and in vivo or to be used as a base for the further improved design of other similar molecules in the search for more efficient and more selective compounds (in silico studies). Some of these approaches, including the anti-inflammatory and antioxidant studies of the series of BTA compounds, are currently under development in our laboratories.

## 3. Materials and Methods

### 3.1. Reagents and Apparatus

All reagents used were commercially obtained from Sigma-Aldrich Chemical Co., Inc. (St Louis, MO, USA), and were used as received without further purification. Solvents were supplied by J.T. Baker (Phillipsburg, NJ, USA), which were dried and distilled prior to use, using standard procedures established under dinitrogen atmosphere. The melting points were determined and are reported without correction using a MELT-TEMP II Laboratory Devices, and vibrational spectroscopy IR was performed in the range of 4000 to 350 cm^−1^ in a NICOLET MAGNA spectrometer 750 FT-IR in KBr discs. MS-ESI were carried out using a JEOL JMS-SX102A spectrometer. NMR spectra were recorded in DMSO-*d*_6_ at room temperature on a JEOL spectrometer GX300 ECLIPSE with 300 Hz frequency for ^1^H, 75 Hz for ^13^C{^1^H}, 282 Hz for ^19^F{^1^H}. The chemical shifts (*δ*) for ^1^H and ^13^C{^1^H} are reported in ppm at low field in relation to TMS or the residual signal of the solvents employed. In the case of the ^19^F{^1^H}, F_3_CCO_2_H was used as external reference. All reactions were performed in open atmosphere.

### 3.2. Synthesis of 2-benzylsulfanyl BTAs Derivatives (1–7)

The BTA derivatives were obtained by the following general procedure: A suspension of 2-mercaptoBTA (1.0 eq) and K_2_CO_3_ (1.1 eq) in THF, where the corresponding benzyl chloride (1.1 eq) was added dropwise and, thereafter, a catalytic amount of KI was added. The reaction mixture was stirred at room temperature for 24 h. The course of the reaction was monitored by thin-layer chromatography. Over time, the reaction mixture was filtered and washed with CH_2_Cl_2_ (3 × 20 mL). The organic filtrate was washed with brine (20 mL), dried with anhydrous Na_2_SO_4_, and evaporated in vacuo to give solids, in most of cases, which were purified by recrystallization from hexane: AcOEt 9:1 to afford amorphous solids.

*2-(benzylthio)benzo[d]thiazole* (**3a**). Light yellow amorphous solid (1.5 g, 5.8 mmol, 75%), mp 34–36 °C. IR (KBr), *ν* (cm^−1^): 3106, 3083, 3056, 3030, 2926, 2838, 1898, 1817, 1688, 1584, 1558, 1492, 1453, 1421, 1307, 1274, 1236, 1190, 1120, 1074, 994, 933, 916, 889, 858, 811, 768, 748, 706, 673, 620. MS-ESI, *m/z*: 258 (100, [M^+^ + 1]). ^1^H NMR (300 MHz, DMSO-*d_6_*), *δ* (ppm): 8.14 (d, *J* = 7.9 Hz, 1H, Ar), 8.04 (d, *J* = 8.1 Hz, 1H, Ar), 7.50–7.45 (m, 3H, Ar), 7.37–7.32 (m, 3H, Ar), 7.29–7.26 (m, 1H, Ar), 4.80 (s, 2H, CH_2_). ^13^C{^1^H} NMR (75 MHz, DMSO-*d_6_*), *δ* (ppm): 166.03, 152.56, 136.47, 134.62, 128.99, 128.50, 127,52, 126.32, 124.44, 121.72, 121.13, 36.57. Elem. Anal. Anal. Calc. for C_14_H_11_NS_2_ (257.37 gmol^−1^): C, 65.33; H, 4.31; N, 5.44; S, 24.92 Found: C 64.83, H 4.29, N, 5.40; S 24.46.

*2-(4-chlorobenzylthio)benzo[d]thiazole* (**3b**). Yellowish amorphous solid (1.2 g, 4.11 mmol, 80%), mp 78–80 °C. IR (KBr), ν (cm^−1^): 3060, 2923, 1589, 1555, 1487, 1453, 1421, 1307, 1273, 1235, 1187, 1120, 1088, 997, 939, 892, 862, 828, 805, 751, 726, 701, 679, 645. MS-ESI *m/z*: 292 (100, [M^+^ + 1]), 294 (45, [M^+^ + 2]), 295 (10), 258 (20),125 (12). ^1^H NMR (300 MHz, DMSO-*d_6_*) *δ* (ppm): 7.84 (d, *J* =8.0 Hz, 1H, Ar), 7.75 (d, *J* = 8.1 Hz, 1H, Ar), 7.38 (d, *J* = 6.8 Hz, 1H, Ar), 7.33 (t, *J* = 7.2 Hz, 1H, Ar), 7.25-7.20 (m, 3H, Ar), 4.63 (s, 2H, CH_2_). ^13^C{^1^H} NMR (75 MHz, DMSO-*d_6_*), *δ* (ppm): 165.77, 152.52, 136.86, 134.69, 132.17, 131.91, 128.46, 126.38, 124.54, 122.79, 121.22, 36.71. Elem. Anal. Anal. Calc. for C_14_H_10_Cl_1_N_1_S_2_ (291.82 gmol^−1^): C, 57.62; H, 3.45; N, 4.80; S, 21.98 Found: C 57.80, H 3.42, N, 4.68; S 22.00.

*2-(4-methoxybenzylthio)benzo[d]thiazole* (**3c**). Yellow amorphous solid (1.17 g, 4.07 mmol, 80%), mp 63–65 °C. IR (KBr), ν (cm^−1^): 3050, 3009, 2949, 2930, 2903, 2832, 1985, 1878, 1724, 1610, 1581, 1507, 1456, 1424, 1301, 1279, 1235, 1197, 1174, 1124, 1093, 1071, 1025, 995, 935, 898, 823, 752, 698. MS-ESI *m/z*: 288 (30, [M^+^ + 1]), 121 (100). ^1^H NMR (300 MHz, DMSO-*d_6_*) *δ* (ppm): 8.00 (d, *J* =7.9 Hz, 1H, Ar), 7.89 (d, *J* = 8.1 Hz, 1H, Ar), 7.50–7.33 (m, 4H, Ar), 6.9 (d, *J* = 8.6 Hz, 2H, Ar), 4.72 (s, 2H, CH_2_), 3.86 (s, 3H, OCH_3_). ^13^C{^1^H} NMR (75 MHz, DMSO-*d_6_*), *δ* (ppm): 166.22, 158.72, 152.62, 134.62, 130.33, 128.11, 126.34, 124.45, 121.75, 121.14, 113.95, 55.04, 36.36. Elem. Anal. Anal. Calc. for C_15_H_13_N_1_O_1_S_2_ (287.4 gmol^−1^): C, 62.69; H, 4.56; N, 4.87; S, 22.31 Found: C 62.57, H 4.54, N, 4.74; S 22.13.

*2-(4-fluorobenzylthio)benzo[d]thiazole* (**3d**). Yellowish amorphous solid (1.47 g, 6.24 mmol, 73%), mp 61–63 °C. IR (KBr), ν (cm^−1^): 3429, 3064, 2928, 2850, 1735, 1596, 1503, 1453, 1422, 1370, 1308, 1270, 1243, 1219, 1154, 1122, 1108, 1077, 1054, 1016, 990, 940, 899, 832, 751, 725, 704, 690, 668. MS-EI *m/z*: 275(38, [M^+^ + 1]), 109 (100), 242 (20), 179 (2), 166 (5), 139 (2), 122 (3), 83 (12), 69 (3), 63(3), 39(3). ^1^H NMR (300 MHz, DMSO-*d_6_*) *δ* (ppm): 8.10 (d, *J* =7.9 Hz, 1H, Ar), 8.0 (d, *J* = 8.1 Hz, 1H, Ar), 7.68-7.63 (m, Ar), 7.58 (t, Ar), 7.47 (t, *J* = 7.5 Hz, 1H, Ar), 7.27 (t, *J* = 8.7 Hz, 2H, Ar), 4.75 (s, 2H, CH_2_). ^13^C{^1^H} NMR (75 MHz, DMSO-*d_6_*), *δ* (ppm):165.94, 161.54 (d, *J* = 243.9 Hz), 152.58, 134.70, 132.92 (d, *J* = 3.1 Hz), 131.12 (d, *J* = 8.3 Hz), 126.40, 124.54, 121.80, 121.20, 115.36 (d, *J* = 29.5 Hz), 35.74. ^19^F{^1^H} NMR (282 MHz, DMSO-*d_6_*) δ (ppm): -114.58. Elem. Anal. Anal. Calc. for C_14_H_10_F_1_N_1_S_2_ (275.36 gmol^−1^): C, 61.06; H, 3.66; N, 5.09; S, 23.29 Found: C 61.02, H 3.58, N, 5.11; S 23.23.

*2-(3-fluorobenzylthio)benzo[d]thiazole* (**3e**). Amber liquid (1.25 g, 4.54 mmol, 83%). IR (KBr), ν (cm^−1^): 3060, 2931, 1738, 1614, 1587, 1486, 1453, 1424, 1307, 1256, 1236, 1135, 1074, 993, 942, 879, 784, 751, 723, 707, 678. MS-ESI *m/z*: 276(100, [M^+^ + 1]), 277 (18), 278 (10). ^1^H NMR (300 MHz, DMSO-*d_6_*) *δ* (ppm): 8.00 (d, *J* = 7.9 Hz, 1H, Ar), 7.90 (d, *J* = 8.1 Hz, 1H, Ar), 7.47 (t, *J* = 7.6 Hz, 1H, Ar), 7.36 (t, 4H, Ar), 7.11 (t, *J* = 7.9 Hz, 1H, Ar), 4.77 (s, 2H, CH_2_). ^13^C{^1^H} NMR (75 MHz, DMSO-*d_6_*), *δ* (ppm):165.79, 162.02 (d, *J* = 243.9 Hz), 152.54, 140.11 (d, *J* = 7.7 Hz),134.73, 130.95 (d, *J* = 8.5 Hz), 126.40, 125.19, 124.55, 121.81, 121.20, 116.30 (d, *J* = 21.9 Hz), 114.90 (d, *J* = 20.9 Hz), 33.59. ^19^F{^1^H} NMR (282 MHz, DMSO-*d_6_*) *δ* (ppm): −112.97. Elem. Anal. Anal. Calc. for C_14_H_10_F_1_N_1_S_2_ (275.36 gmol^−1^): C, 61.06; H, 3.66; N, 5.09; S, 23.29 Found: C 60.98, H 3.63, N, 5.01; S 23.15.

*2-(2,6-difluorobenzylthio)benzo[d]thiazole* (**3f**). Amber liquid (1.152 g, 3.93 mmol, 77%). IR (KBr), ν (cm^−1^): 3376, 3062, 3000, 2953, 1738, 1623, 1589, 1561, 1509, 1465, 1424, 1375, 1308, 1272, 1236, 1164, 1131, 1074, 993, 936, 853, 826, 786, 753, 725, 702, 661. MS-ESI *m/z*: 294 (100, [M^+^ + 1]), 295 (42), 296 (25). ^1^H NMR (300 MHz, DMSO-*d_6_*) *δ* (ppm): 8.14 (d, *J* =7.8 Hz, 1H, Ar), 8.02 (d, *J* = 8.0 Hz, 1H, Ar), 7.63–7.48 (m, 3H, Ar), 7.26 (t, *J* = 8.0, 2H, Ar), 4.81 (s, 2H, CH_2_). ^13^C{^1^H} NMR (75 MHz, DMSO-*d_6_*), *δ* (ppm):164.73, 160.62 (dd, *J* = 249.8, 8.8 Hz), 152.54, 134.96,131.05 (t, *J* = 10.4 Hz), 126.50, 124.80, 121.93, 112.27 (m), 24.49. ^19^F{^1^H} NMR (282 MHz, DMSO-*d_6_*) *δ* (ppm): −113.36. Elem. Anal. Anal. Calc. for C_14_H_9_F_2_N_1_S_2_ (293.35 gmol^−1^): C, 57.32; H, 3.09; N, 4.97; S, 23.08 Found: C 57.13, H 3.02, N, 5.01; S 23.15.

*2-(perfluorobenzylthio)benzo[d]thiazole* (**3g**). Yellowish amorphous solid (1.298 g, 3.73 mmol, 86%), mp 54–56 °C. IR (KBr), ν (cm^−1^): 3065, 2963, 1737, 1654, 1501, 1454, 1423, 1306, 1274, 1237, 1169, 1123, 1078, 1038, 985, 967, 882, 756, 726, 703, 682, 640, 603. MS-ESI *m/z*: 348(100, [M^+^ + 1]), 349 (45), 350 (25). ^1^H NMR (300 MHz, DMSO-*d_6_*) *δ* (ppm): 8.04 (d, *J* = 7.90 Hz, 1H, Ar), 7.87 (d, *J* = 8.1 Hz, 1H, Ar), 7.50 (t, *J* = 7.60 Hz, 1H, Ar), 7.40 (t, *J* = 7.6, 1H, Ar), 4.77 (s, 2H, CH_2_). ^13^C{^1^H} NMR (75 MHz, DMSO-*d_6_*), *δ* (ppm):164.01, 152.37, 144.84 (dt, *J* = 244.2, 13.5 Hz), 140.15 (dt, *J* = 248.8, 13.5 Hz), 136.90 (dt, *J* = 249.9, 15.8 Hz), 135.01, 126.53, 124.88, 122.00, 121.35, 111.36 (t, *J* = 19.5 Hz), 24.21. ^19^F{^1^H} NMR (282 MHz, DMSO-*d_6_*) *δ* (ppm): −140.92 (dd, *J* = 22.8, 7.2 Hz), −154.91, -155.11 (m), −162.54–162.71 (m). Elem. Anal. Anal. Calc. for C_14_H_6_F_5_N_1_S_2_ (347.33 gmol^−1^): C, 48.41; H, 1.74; N, 4.03; S, 18.46 Found: C 48.37 H 1.67, N, 4.05; S 18.50.

### 3.3. Biological Activity

#### 3.3.1. Trypanocidal Activity Evaluation

Compounds and reference drugs, nifurtimox (Nfx: Lampit™, Bayer) and Bnz (Rochagan™, Roche), were dissolved at 10 mg/mL in dimethyl sulfoxide (DMSO, Sigma-Aldrich), and corresponding dilutions were carried out in phosphate-buffered saline (PBS).

Bloodstream trypomastigotes of two strain of *T. cruzi* were used: NINOA strain (MHOM/MX/1994/NINOA, isolated from a patient in acute phase in Oaxaca, Mexico) and INC-5 strain (MHOM/MX/1994/INC5, isolated from a patient in chronic phase in Guanajuato, Mexico). CD1 mice 6–8 weeks old were intraperitoneally infected; at the maximum peak of parasitaemia (4 weeks), infected blood was obtained by cardiac puncture using heparin as anticoagulant. Infected blood was adjusted to 1 × 10^6^ parasites/mL and seeded into 96-well microplates. Animal experiments were performed according to Norma Oficial Mexicana (NOM-062-Z00-1999) published on august 22, 2009 entitled Technical specifications for the production, care and use of laboratory animals. The compounds were evaluated at different concentrations obtained by serial dilutions starting from 100 µg/mL. In each well, 90 μL of infected blood and 10 μL of the corresponding compound or reference drug were deposited. A positive control of lysis reference drugs were used, and DMSO 1% was used as the negative control. All assays were performed in triplicate. The microplate was incubated 24 h at 4 °C.

The quantification of bloodstream trypomastigotes was performed by the Brener-Pizzi method, 5 μL of blood was deposited between a slide and a coverslip (18 × 18 mm), and all bloodstream trypomastigotes present in 20 fields were quantified in an optical microscope at 40×. The amount of trypomastigotes from each sample was compared to the negative control and the percentage of lysis was determined; finally, the lytic concentration of 50% parasites (LC_50_) were calculated for each compound and converted to micromolar data [23].

#### 3.3.2. Cytotoxic Activity Evaluation and SI

For this assay, murine macrophage cell line J774A.1 (TIB-61 ATCC) were used in RPMI 1640 medium (Gibco, Carlsbad, CA, USA), enriched with 10% inactivated fetal bovine serum (FBS, Gibco Carlsbad, CA, USA), 1% penicillin-streptomycin (In vitro S.A., Mexico City, Mexico) and 1% MEM-NEA medium (Gibco, Carlsbad, CA, USA), the cells were kept at 37 °C with 5% CO_2_ and moisture atmosphere. Initially, on a 96-well microplate, they were placed 5 × 10^4^ per well and incubated for 24 h. The compounds were tested at different concentrations, starting from 100 µg/mL and by triplicate, as cytotoxicity negative control untreated cells were used. The microplate was incubated for 20 h under the same conditions; afterward, 10 µL of resazurin (Invitrogen, Grand Island, NY, USA) 0.01% was added and incubated for an additional 4 h period. The microplate was analyzed on a fluorometer at 544 nm excitation/590 nm emission (Spectramax Plus; Molecular Devices, Sunnyvale, CA, U.S.). As a negative control of cytotoxicity, macrophages treated with 0.1% DMSO were used. The cytotoxic concentration of 50% of population (CC_50_) was determined using the de Probit statistical tool and converted to micromolar data. Finally, the ratio between CC_50_ of macrophages and LC_50_ of the bloodstream trypomastigotes (CC_50_/ CL_50_) was calculated to get SI values [23].

## Figures and Tables

**Table 1:** Structures and yields of synthesized BTA derivatives.

Entry	Benzyl chloride	Compound	Yield ^a^ (%)
1	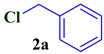	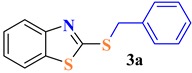	75
2	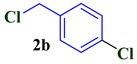	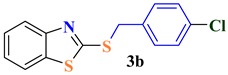	80
3	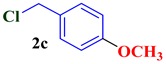	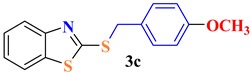	80
4	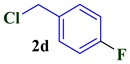	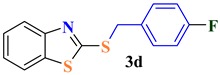	73
5	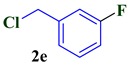	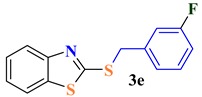	83
6	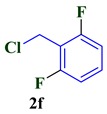	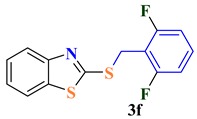	77
7	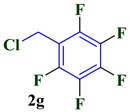	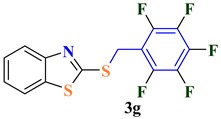	87

^a^ Isolated yield based on 2-mercaptoBTA.

**Table 2:** Biological activity of BTA derivatives on *T. cruzi* NINOA and INC-5 strain.

*Compound*	Bloodstream Trypomastigotes LC_50_ (µM)		Macrophages J774A.1 CC_50_ (µM)	SI (CC_50_ /LC_50_)	
	*T. cruzi* *NINOA*	*T. cruzi* *INC-5*		*T. cruzi* *NINOA*	*T. cruzi* *INC-5*
3a	123.44 ± 21.86	335.59 ±35.7	>900	>7.29	>2.68
3b	>350	262.62 ± 12.33	>900	Nd	>3.42
3c	307.82 ± 28.88	275.67 ± 22.96	>900	>2.92	>3.26
3d	>350	>350	869.91 ± 26.8	Nd	Nd
3e	>350	>350	784.82 ± 25.45	Nd	Nd
3f	109.76 ± 23.18	259.81 ± 25.56	491.88 ± 10.09	4.48	1.91
3g	146.95 ± 21.01	185.35 ± 12.95	249.56 ± 9.44	1.69	1.34
*Nfx*	96.26 ± 11.48	127.63 ±14.97	347.32 ± 18.26	3.61	2.72
*Bnz*	173.46 ± 15.89	216.57 ± 23.08	223.43 ± 11.23	1.28	1.05
Nd: Not determined.					
